# Low frequency of flares during pregnancy and post-partum in stable lupus patients

**DOI:** 10.1186/s13075-020-2139-9

**Published:** 2020-03-19

**Authors:** Julia Davis-Porada, Mimi Y. Kim, Marta M. Guerra, Carl A. Laskin, Michelle Petri, Michael D. Lockshin, Lisa R. Sammaritano, D. Ware Branch, Allen Sawitzke, Joan T. Merrill, Jill P. Buyon, Jane E. Salmon

**Affiliations:** 1 Hospital for Special Surgery, 535 E 70th Street, New York, NY 10021 USA; 2 Albert Einstein College of Medicine, New York, NY USA; 3 Mount Sinai Hospital Toronto, Toronto, Ontario Canada; 4 Trio Fertility, Toronto, Ontario Canada; 5 Johns Hopkins University School of Medicine, Baltimore, MD USA; 6 Weill Cornell Medicine, New York, NY USA; 7 Intermountain Healthcare, Salt Lake City, UT USA; 8 University of Utah Health Sciences Center, Salt Lake City, UT USA; 9 University of Oklahoma Health Sciences Center, Oklahoma City, OK USA; 10 Oklahoma Medical Research Foundation, Oklahoma City, OK USA; 11 New York University School of Medicine, 550 1st Avenue, New York, NY 10016 USA

**Keywords:** SLE, Pregnancy, Flares

## Abstract

**Background:**

Lupus patients are at risk for pregnancy loss, and it has been generally accepted that women with SLE should have low disease activity prior to conception. However, there are conflicting results regarding the effect of pregnancy on SLE flares. This study aims to identify predictors of flares during and after pregnancy in SLE patients with inactive or stable disease activity during the first trimester and to characterize and estimate the frequency of post-partum flares in these patients.

**Methods:**

SLE patients in the multicenter, prospective PROMISSE (Predictors of Pregnancy Outcome: Biomarkers in Antiphospholipid Antibody Syndrome and Systemic Lupus Erythematosus) study were evaluated for flares during and after pregnancy using the SELENA-SLEDAI Flare Index. Flares during pregnancy were assessed in all 384 patients and post-partum flares in 234 patients with study visits 2–6 months post-partum. Logistic regression models were fit to the data to identify independent risk factors for flare.

**Results:**

During pregnancy, 20.8% of patients had mild/moderate flares and 6.25% had severe. Post-partum, 27.7% of patients had mild/moderate flares and 1.7% had severe. The mild flares rarely required treatment. Younger age, low C4 and higher PGA at baseline were independently associated with higher risk of having at least one mild/moderate or severe flare during pregnancy. Older patients were at decreased risk of flare, as well as those with quiescent disease at baseline. No variables evaluated at baseline or the visit most proximal to delivery was significantly associated with risk of flare post-partum. Medications were not associated with flare during or after pregnancy.

**Conclusion:**

In patients with inactive or stable mild disease activity at the time of conception, lupus disease flares during and after pregnancy are typically mild and occur at similar rates. Flares during pregnancy are predicted by the patients’ age and clinical and serological activity at baseline.

## Background

Systemic lupus erythematosus (SLE) primarily affects women of childbearing age. Lupus patients are at increased risk for poor fetal outcomes, including pre-term birth, growth restriction, and fetal loss, and for poor maternal outcomes, including nephritis and preeclampsia [1–3]. Active disease is a predictor of these poor outcomes; thus, current clinical guidance is to conceive at a time following low disease activity for 6 months [2, 4]. However, there is no consensus on the effect of pregnancy on disease activity during pregnancy and the postpartum period. What predicts disease flares during these periods remains an unanswered question.

Reported rates of flare during pregnancy vary widely, from 13 to 74% [5–7]. Previous studies in pregnant SLE patients have included those with high and low disease activity at baseline. In prospective studies, rates of flare during pregnancy range from 0.6 to 1.68 per person-year and from 0.36 to 1.8 per person-year post-partum [8–12].

We previously reported that in the PROMISSE study, a prospective, multiracial, multiethnic cohort of pregnant SLE patients with inactive or stable mild disease activity at baseline (8–12 weeks gestation), 12.7% of patients had mild/moderate flares during the second trimester and 9.6% during the third trimester. 2.5% of patients had severe flares during the second trimester and 3.0% in the third, mostly consisting of nephritis, pleuritis, and arthritis [1]. These rates are relatively low, and most mild/moderate flares required no treatments.

The objective of the current study is to determine the rate and characteristics of post-partum flares and to identify independent predictors of experiencing any type of flare during pregnancy and post-partum. We also estimated the incidence of flare per person-year to compare our results to the only other large, multiracial, prospective cohort of SLE pregnancy [9, 10]. We employed data from the PROMISSE (Predictors of Pregnancy Outcome: Biomarkers in Antiphospholipid Antibody Syndrome and Systemic Lupus Erythematosus) study, a prospective, multiethnic, multiracial cohort of SLE patients.

## Methods

### Study design

PROMISSE study enrolled pregnant women with SLE meeting the revised American College of Rheumatology criteria, women with antiphospholipid antibodies (aPLs), and healthy women at 8–12 weeks gestation and followed them monthly and approximately 3 months post-partum [1, 13]. The current report focuses on the patients with SLE with or without aPLs.

### Patient population

Between September 2003 and December 2012, consecutive pregnant patients meeting inclusion criteria (age 18 to 45 years with hematocrit greater than 26% were enrolled at less than 12 weeks gestation) were enrolled at 8 sites in the USA and 1 in Canada. The PROMISSE study was designed to identify predictors of adverse pregnancy outcomes in patients with SLE. To restrict our analysis to patients with quiescent or stable, mildly active disease and avoid other known risk factors of pregnancy complications that may be present in SLE patients with active disease, patients taking prednisone greater than 20 mg per day, with proteinuria greater than 1 g/24 h, or creatinine greater than 1.2 mg/dL were excluded. All baseline clinical and laboratory measures were collected at the screening visit (8–12 weeks gestation). All women with SLE enrolled in PROMISSE (*n* = 384) were included in the intrapartum analysis. In the analysis of post-partum flares, we included patients who had study visits between 2 and 6 months post-partum (*n* = 234) to include the maximum number of patients where disease activity could be attributable to the post-partum state. Most of the other 150 patients presented for their post-partum visits well beyond 6 months, and active SLE, if present, would not likely be related to an effect conferred by pregnancy per se.

### Definition of SLE disease activity and flares during and after pregnancy

Disease activity was measured at each visit using the Systemic Lupus Erythematosus Pregnancy Disease Activity Index (SLEPDAI) and Physician Global Assessment (PGA). This instrument has been modified from the SELENA-SLEDAI to discount changes of pregnancy that mimic lupus disease activity, such as chloasma and headaches associated with PE [14]. Flares were defined using the SELENA-SLEDAI flare Index and were assessed during each trimester and at 2 to 6 months post-partum. If a patient had a flare between trimester visits, this was recorded in an interim visit or by review of medical records if seen by another physician and incorporated into our results. Study investigators initially were trained with “paper” pregnant patients with SLE and interrater reliability in the assessment of flares was subsequently evaluated. As previously reported, the average correlation of investigator responses with the gold standard response was 0.89 (95% CI, 0.83–0.95), and the inter-rater reliability estimated by the intraclass correlation coefficient was 0.78 (95% CI, 0.61–0.89) [1]. In some cases if the patients were seen by a rheumatologist other than the study investigator during a flare, medical records from that physician were reviewed.

### Statistical analysis

The flare data were summarized by computing the proportion of patients who experienced any flare during the relevant time period (pregnancy, post-partum), as well as the incidence of flare per person-month of follow-up which included data on all occurrences of flares from all patients. Bivariate associations of baseline patient characteristics and flare status (i.e., whether patient experienced any flare) during and after pregnancy were evaluated using the *T* test for continuous variables and the chi-square test for categorical variables. Multivariable analyses were conducted by fitting logistic regression models to identify independent predictors of having at least one flare during pregnancy. A step-wise variable selection approach was applied, and the final logistic regression model included only those predictors that remained significant at the *p* < 0.05 level. To account for multiple occurrences of flare in the same patient, a negative binomial regression model using the logarithm of the gestational age as the offset term was also fit to the data. As previously reported, missing data rates for all baseline variables were low (0–7%), with the highest missing data rates occurring for C4 (7%), C3 (5%), PGA (5%), and urinary protein excretion (3%) [1]. Missing data were handled using both listwise deletion and multiple imputation using chained equations; results were nearly identical so only those based on the former are reported.

## Results

The SLE patients had an average age of 30.9 years and inactive or mild stable disease activity at baseline with a mean SLEPDAI score of 2.79 (SD 2.99) and mean PGA of 0.4 (SD 0.54) (Table 1). Among the 234 patients who had their post-partum visits between 2 and 6 months after delivery, 191 patients remained pregnant beyond 36 weeks, 22 delivered between 32 and 36 weeks, 16 between 22 and 32 weeks, and 5 by 20 weeks.

**Table 1:** Association between baseline patient characteristics and flare status

Characteristic	During pregnancy				Post-partum			
	Total, ***n*** = 384	No flare, ***n*** = 284	Flare, ***n*** = 100	***p*** value	Total, ***n*** = 234	No flare, ***n*** = 177	Flare, ***n*** = 57	***p*** value
Demographic characteristics								
Ethnicity/race				0.019				0.85
Non-Hispanic White	188 (49)	149 (52.5)	39 (39)		118 (50.4)	90 (50.8)	28 (49.1)	
Hispanic White	30 (7.8)	19 (6.7)	11 (11)		15 (6.4)	13 (7.3)	2 (3.5)	
African American	78 (20.3)	53 (18.7)	25 (25)		42 (17.9)	30 (16.9)	12 (21.1)	
Asian	41 (10.7)	34 (12.0)	7 (7)		32 (13.7)	25 (14.1)	7 (12.3)	
Other	10 (2.6)	8 (2.8)	2 (2)		7 (3)	5 (2.8)	2 (3.5)	
Unknown	37 (9.6)	21 (7.4)	16 (16)		20 (8.5)	14 (7.9)	6 (10.5)	
Mean age (SD), years	30.92 (4.89)	31.4 (4.73)	29.56 (5.08)	0.002	31.19 (4.48)	31.24 (4.39)	31.04 (4.78)	0.78
Ever lupus nephritis				0.75				0.53
No	264 (68.8)	197 (69.4)	67 (67)		160 (68)	123 (69)	37 (65)	
Yes	120 (31.2)	87 (30.6)	33 (33)		74 (32)	54 (41)	20 (35)	
Laboratory values								
Anti dsDNA antibodies				0.85				0.47
Negative	217 (58)	162 (58.5)	55 (56.7)		132 (64)	98 (62)	34 (69)	
Positive	157 (42)	115 (41.5)	42 (43.3)		74 (36)	59 (38)	15 (31)	
aPL (LAC, aβ2GP1, aCL)				0.25				0.15
Negative	322 (83.9)	234 (82.4)	88 (88)		195 (83)	144 (81)	51 (89)	
Positive	62 (16.1)	50 (17.6)	12 (12)		39 (17)	33 (19)	6 (11)	
LAC				0.17				0.64
Negative	350 (91.1)	255 (89.8)	95 (95)		213 (91)	162 (92)	51 (89)	
Positive	34 (8.9)	29 (10.2)	5 (5)		21 (9)	15 (8)	6 (11)	
Urinary protein excretion > 500 mg/day				1				1
No	338 (91.1)	251 (91.3)	87 (90.6)		201 (89)	153 (89)	48 (89)	
Yes	33 (8.9)	24 (8.7)	9 (9.4)		24 (11)	18 (11)	6 (11)	
Platelet count								
Mean (SD), × 10^6^ cells/L	253.1 (84.32)	252.96 (84.25)	253.39 (84.94)	0.97	234.56 (75.25)	236.41 (76.23)	228.73 (72.49)	0.52
< 100 × 10^6^ cells/L				0.054				
No	369 (97.6)	276 (98.6)	93 (94.9)					
Yes	9 (2.4)	4 (1.4)	5 (5.1)					
Low complement				0.021				0.61
No	251 (65.9)	195 (69.4)	56 (56)		155 (74)	120 (75)	35 (70)	
Yes	130 (34.1)	86 (30.6)	44 (44)		55 (26)	40 (25)	15 (30)	
Mean C3 (SD) g/L	104.57 (27.15)	105.06 (26.15)	103.17 (29.91)	0.56	121.86 (28.55)	120.75 (28.20)	125.37 (29.65)	0.34
C3 low or normal				0.10				
Normal	291 (80)	221 (82.2)	70 (73.7)					
Low	73 (20)	48 (17.8)	25 (26.3)					
Mean C4 (SD), g/L	19.84 (8.28)	20.3 (8.46)	18.40 (7.68)	0.051	21.07 (9.23)	21.36 (9.36)	20.20 (8.87)	0.43
C4 low or normal				0.005				
Normal	266 (74.1)	207 (78.1)	59 (62.8)					
Low	93 (25.9)	58 (21.9)	35 (37.2)					
Disease activity								
SLEPDAI								
Mean score (SD)	2.79 (2.99)	2.55 (2.89)	3.47 (3.18)	0.012	2.09 (2.76)	2.22 (2.76)	1.68 (2.76)	0.22
SLEPDAI > 4				0.015				0.38
No	310 (81.6)	237 (84.6)	73 (73)		192 (88)	143 (87)	49 (92)	
Yes	70 (18.4)	43 (15.4)	27 (27)		26 (12)	22 (13)	4 (8)	
PGA								
Mean score (SD)	0.4 (0.54)	0.33 (0.5)	0.59 (0.6)	*p* < 0.001	0.35 (0.5)	0.34 (0.5)	0.39 (0.52)	0.49
PGA > 1				0.005				0.55
No	327 (89.3)	249 (92.2)	78 (81.2)		201 (93)	155 (94)	46 (90)	
Yes	39 (10.7)	21 (7.8)	18 (18.8)		15 (7)	10 (6)	5 (10)	
Current medications								
Glucocorticoids				0.25				1
No	230 (59.9)	175 (61.6)	55 (55)		136 (58)	103 (58)	33 (58)	
Yes	154 (40.1)	109 (38.4)	45 (45)		98 (42)	74 (42)	24 (42)	
Hydroxychloroquine				0.79				0.23
No	136 (35.4)	99 (34.9)	37 (37)		79 (34)	56 (32)	23 (40)	
Yes	248 (64.6)	185 (65.1)	63 (63)		155 (66)	121 (68)	34 (60)	
Azathioprine				0.41				0.52
No	314 (81.8)	229 (80.6)	85 (85)		186 (79)	139 (79)	47 (82)	
Yes	70 (18.2)	55 (19.4)	15 (15)		48 (21)	38 (21)	10 (18)	
Heparin				0.34				0.42
No	300 (78.1)	218 (76.8)	82 (82)		184 (79)	137 (77)	47 (82)	
Yes	84 (21.9)	66 (23.2)	18 (18)		50 (21)	40 (23)	10 (18)	
Aspirin				0.17				0.24
No	249 (64.8)	178 (62.7)	71 (71)		162 (69)	119 (67)	43 (75)	
Yes	135 (35.2)	106 (37.3)	29 (29)		72 (31)	58 (33)	14 s(25)	
Anti-hypertensives				1				0.44
No	351 (91.4)	260 (91.5)	91 (91)		215 (92)	164 (93)	51 (89)	
Yes	33 (8.6)	24 (8.5)	9 (9)		19 (8)	13 (7)	6 (11)	
Flares during pregnancy	–	–	–	–				0.69
No	–	–	–		169 (72)	129 (73)	40 (70)	
Yes	–	–	–		65 (28)	48 (27)	17 (30)	

### Rates of flares

One hundred patients out of 384 (26%) flared at any point during pregnancy; 20.8% of patients had at least 1 mild/moderate flare, and 6.25% had at least 1 severe flare. The overall incidence rates of flare during pregnancy were 0.3 per person-year for mild/moderate flares and 0.09 per person-year for severe flares. The majority of all flares occurred in the second and third trimesters, with only 4% occurring in the first trimester.

In the post-partum period, defined as 2–6 months after the end of pregnancy, most patients had quiescent disease; only 23 of 234 patients (9.8%) had a SLEPDAI score > 6, and only 20 patients (8.4%) had a PGA > 1 (Table 2). Mild/moderate flares were documented in 22.7% of patients and severe flares in 1.7%, similar to the rates observed during pregnancy. Flares were distributed throughout the post-partum period: 22.8% after 2 months, 45.6% after 3 months, 21.1% after 4 months, and 10.5% after 5 months. Post-partum incidence rates for mild/moderate and severe flares were 0.8 and 0.06 per person-year, respectively. The flares were mild and typically comprised serologic or cutaneous manifestations (e.g., newly positive anti-dsDNA and/or low complements). Serious visceral complications were rare (Table 2). Of the patients with increased post-partum proteinuria (> 0.5 g/24 h change or with a change in urine dipstick by 2), 11 of the 12 had a history of lupus nephritis prior to their pregnancies, and only one had a change in therapy. The patient with vasculitis had low-grade vasculitis typical for that patient, and the patient with psychosis did not have focal neurologic findings and had intermittent psychotic thoughts. Only 19 of 57 (33.3%) postpartum flares were treated: 13 with an increase in prednisone, 6 with NSAID or hydroxychloroquine, and 1 with mycophenolate mofetil, which was started in a patient with a severe flare and persistent proteinuria (Table 1). Otherwise, consequences of post-partum flares were minimal.

**Table 2:** Characteristics of post-partum flare (by definition all domains were newly scored as positive)

Present SLEPDAI characteristic	Total (***n*** = 57 flares)
Positive anti-dsDNA	25 (43.9%)
Low complement	20 (35.1%)
Rash	20 (35.1%)
Arthritis	14 (24.6%)
Proteinuria	12 (21.1%)
Mucosal ulcers	9 (15.8%)
Alopecia	4 (7.0%)
Pyuria	3 (5.3%)
Leukopenia	2 (3.5)
Hematuria	1 (1.8%)
Pleurisy	1 (1.8%)
Pericarditis	1 (1.8%)
Vasculitis	1 (1.8%)
Myositis	1 (1.8%)
Psychosis	1 (1.8%)
Physician-initiated changes	
Increase in prednisone but not to > 0.5 mg/kg/day	13 (22.8%)
Increase in prednisone to > 0.5 mg/kg/day	0 (0%)
> 1.0 increase in PGA, but not to > 2.5	6 (10.5%)
Added NSAID or hydroxychloroquine for disease activity	6 (10.5)
Added mycophenolate mofetil	1 (1.8%)

### Bivariate analyses of risk factors for flare

We evaluated the association of baseline characteristics (collected at < 12 weeks gestation), laboratory values, disease, activity, and medications with occurrence of flare of any severity during pregnancy (Table 1). Baseline demographic and clinical variables that were significantly associated with experiencing a flare during pregnancy were ethnicity/race (*p* = 0.019) and age (*p* = 0.002). Patients who did not have flares during pregnancy were more likely to be non-Hispanic white and older. Of laboratory values, low C4 was significantly associated with flare (*p* = 0.005). Disease activity at baseline was higher in patients who had a flare during pregnancy. Specifically, mean SLEPDAI (*p* = 0.012), mean PGA (*p* < 0.001), proportions of patients with SLEPDAI greater than 4 (*p* = 0.011), and proportion with PGA greater than 1 (*p* = 0.005) were all higher in the group that flared. None of the medications evaluated, including glucocorticoids, hydroxychloroquine, azathioprine, heparin, aspirin, or anti-hypertensives, were significantly associated with flare during pregnancy.

In contrast to the findings observed for flares that occurred during pregnancy, baseline patient characteristics were not correlated with post-partum flares. We also evaluated laboratory values, disease activity, and medication use at the last visit during pregnancy and again found that none were associated with post-partum flares (results not shown).

### Multivariable analyses of predictors of flare during pregnancy

Baseline variables that were identified in a logistic regression model to be independently predictive of having at least one flare during pregnancy included age at screening (*p* = 0.003), low C4 (*p* = 0.024), and PGA score (*p* = 0.0005) (Table 3). Older patients were at decreased risk of experiencing a flare, as well as those with quiescent disease at baseline. Clinical features associated with adverse pregnancy outcome in the PROMISSE study, including platelet count, antihypertensive use, and lupus anticoagulant, were not predictive of flare. When multiple occurrences of flare in the same patient were accounted for in the analysis by fitting a negative binomial regression model to the data, age, low C4, and PGA were again found to be the strongest predictors of flare during pregnancy. Since neither baseline variables nor variables evaluated during the last pregnancy visit were found to be associated with post-partum flares, multivariable analyses of these data were not performed.

**Table 3:** Predictors of flare during pregnancy from multivariable logistic and negative binomial regression analyses

Baseline variable	OR (95% CI)*	*p* value	RR (95% CI)**	*p* value
Age at screening (per increase of 1 year)	0.92 (0.88–0.97)	0.003	0.95 (0.91–0.99)	0.010
Screening C4 (low vs normal)	1.87 (1.08–3.22)	0.024	1.48 (0.97–2.26)	0.068
Screening PGA score (per increase of 0.5)	1.48 (1.19–1.84)	0.0005	1.30 (1.10–1.53)	0.002

*Based on logistic regression model

**Based on negative binomial regression analysis of multiple flares

## Discussion

In this multicenter, multiethnic, and multiracial prospective study of SLE pregnancy, both intrapartum and postpartum flares occurred in a quarter of the patients. The majority of these flares were mild, with few requiring a change in therapy. Patients likely to flare during pregnancy were characterized by higher disease activity at baseline defined by PGA and SLEPDAI scores, non-white race, younger age, and lower complement. However, even in patients with risk factors, rates of flares were low. No predictors of flares post-partum were identified, including having had a flare during pregnancy.

Other studies have assessed lupus flares during pregnancy with differing results. Some report higher rates ranging from 37.7 to 74% of patients [7–12, 15], and several are in accord with our results, showing lower rates of flare (19.4–25%) [2, 5, 6]. In the latter, more than 90% of patients had low, very low, or no disease activity during pregnancy [2, 5, 6]. To compare our study to the only other multi-ethnic prospective cohort, we estimated the incidence of flare per person-year. Mild/moderate flares occurred at a rate of 0.3 per person-year and severe flares at 0.09 per person-year. This rate is lower than that identified in the Hopkins Lupus Cohort of 1.6 flares per person-year reported in 1991 and 0.6 flares per person-year reported in 2018 [9, 10]. Differences between these reports may be accounted for varying definitions of flare applied in each cohort. We selected the SELENA-SLEDAI Flare Index with the adaptation for pregnancy (SLEPDAI). This instrument takes into account attribution to lupus activity and not physiologic changes in pregnancy that might mimic lupus such as chloasma, headache and thrombocytopenia of PE, or hyperemia of hands, and bland knee effusions [14]. The most likely reason for our lower rates of flares is that PROMISSE patients had inactive or stable mild disease activity during the first trimester. Indeed, low flare rates were reported in two studies that included SLE patients with quiescent disease early in their pregnancies. In Cavallasca et al.’s cohort, only 5% of patients had active disease at the beginning of pregnancy, and in Gotestam et al.’s cohort, over 50% of patients had zero disease activity as measured by Lupus Activity Index (LAI) during the first trimester [2, 6]. In contrast, patients in the Hopkins Lupus Cohort in which higher intrapartum rates of flares were observed tended to have more severe disease; 12.5% of patients seen before conception had high disease activity and 10% more patients had a history of nephritis than in our cohort [3, 15]. Of note, only 8% of patients in the Hopkins cohort with low disease activity before pregnancy transitioned to have active lupus during pregnancy compared to 58% of patients who had high disease activity before pregnancy confirming the benefit of quiescent disease before pregnancy [3]. The discrepancy in flare rates among cohorts may also be attributable to differences in the ethnic and racial composition. In support of this consideration, race (non-white) was an independent risk factor for flare in PROMISSE. More than 40% of patients in the Hopkins cohort are black, while in our cohort, only 20% of the patients were black. Other studies that reported lower rates of flare in pregnancy, Lockshin et al. from New York, USA, Cavallasca et al. from Argentina, and Gotestam et al. from Sweden, also had small numbers of black patients [2, 5, 6].

We found no increased rate of lupus flares between 2 and 6 months post-partum compared to that during pregnancy with an overall flare rate of 24.4%; the rate of mild/moderate flares was 0.8 per person- year and the rate of severe flares was 0.06 per person-year. While fewer studies have assessed flares postpartum, our findings are similar to other studies. The highest reported frequency of flare from Ruiz-Irastorza et al. was 1.8 per person-year, suggesting that almost every patient could flare in the first 6 months post-partum [12]. This rate may be much higher than ours and that of the Hopkins cohort (0.36–0.72 flare per person-year), because in this analysis, flares were only in the first 6 weeks post-partum, while we evaluated flares 2–6 months post-partum and the Hopkins cohort assessed flares in the first year post-partum [10]. The Hopkins cohort did find an increase in flare in the initial 12 weeks postpartum, consistent with Ruiz-Irastorza et al. Possibly, this early post-partum period (before 6 weeks) is different from the more extended post-partum period that we evaluated.

In the present study, we found no medications to be associated with flare, including hydroxychloroquine. Other studies have suggested that hydroxychloroquine has a protective effect during pregnancy leading to fewer flares or lower disease activity in patients continuing hydroxychloroquine during pregnancy [2, 10, 15]. While our study found no benefit from hydroxychloroquine, 60% of our patients were taking it throughout pregnancy. Additionally, there may be confounding by indication, and our study was not designed to examine this variable.

Our analysis benefited from studying a large multiethnic, multiracial cohort of patients. Additional strengths are its prospective nature and focus on predictors of flare. However, limitations are acknowledged. This study’s inclusion largely of patients with quiescent disease at baseline and exclusion of those with SLE requiring high dose prednisone and current nephritis limits its generalizability to all SLE patients. Despite the expertise of the rheumatologists involved and careful tracking of patients, it is possible that some flares could have been missed, given scheduling of patients every trimester, although every attempt was made to capture patients when they were experiencing a flare. Scheduling of our post-partum visit at 3 months is a greater limitation, given data from other studies pointing to increased risk soon after birth. Additionally, about 10% of the PROMISSE study patients followed in pregnancy did not have a post-partum visit. Finally, since many patients presented to this study only after conception, we have no data to review disease activity prior to pregnancy to determine whether pregnancy per se increased the risk for flare.

## Conclusions

In patients with inactive or stable mild disease at conception, lupus disease flares during and after pregnancy are typically mild and occur at similar rates. Flares during pregnancy are predicted by age and clinical and serological activity at baseline.

The current study extends previous reports from the PROMISSE study to the post-partum period and examines predictors of flares with multivariate analyses. Based on these results, physicians can reassure their patients that if they plan their pregnancy at a time of quiescence, they are unlikely to have a flare during or in the 6 months after pregnancy.

## Data Availability

The authors declare that the data supporting the findings of this study are available within the article and its supplementary information files.

## Abbreviations

aPLs: Antiphospholipid antibodies
PGA: Physician Global Assessment
PROMISSE: Predictors of Pregnancy Outcome: Biomarkers in Antiphospholipid Antibody Syndrome and Systemic Lupus Erythematosus
SLE: Systemic lupus erythematosus
SLEDAI: Systemic Lupus Erythematosus Disease Activity Index
SLEPDAI: Systemic Lupus Erythematosus Pregnancy Disease Activity Index
